# Elevated retinal artery vascular resistance determined by novel visualized technique of laser speckle flowgraphy in branch retinal vein occlusion

**DOI:** 10.1038/s41598-021-99572-7

**Published:** 2021-10-08

**Authors:** Ryo Tomita, Takeshi Iwase, Marie Fukami, Kensuke Goto, Eimei Ra, Hiroko Terasaki

**Affiliations:** 1 Department of Ophthalmology, Nagoya University Graduate School of Medicine, Nagoya, Aichi 466-8560 Japan; 2 Department of Ophthalmology, Akita University Graduate School of Medicine, 1-1-1 Hondou, Akita, 010-8543 Japan

**Keywords:** Retinal diseases, Eye manifestations

## Abstract

We aimed to investigate the increase in resistivity of the retinal artery in the branch retinal vein occlusion (BRVO)-affected area, and to visualize it. Thirty-two eyes of 32 patients with BRVO were measured by laser speckle flowgraphy (LSFG). The retinal artery and vein running to the BRVO-affected area and vertically symmetrical vessels in the unaffected area were examined. We applied the LSFG parameter beat strength over mean blur rate (BOM), calculated using a similar method to the pulsatility index used in Doppler flowmetry to evaluate resistivity of the vessels. Our results showed that the BOM map could clearly visualize the increase of resistivity in the retinal artery as a two-dimensional map. The BOM of the arteries in the affected area was significantly higher than that of the unaffected area (*P* = 0.001). Multiple regression analysis showed that the ratio of BOM in retinal arteries of the affected area to the unaffected was significantly associated with the extent of retinal hemorrhage (β = 0.447, *P* = 0.009). In conclusion, the index of resistivity of the retinal artery in the BRVO-affected area was higher and could be visualized in a two-dimensional map. These findings and techniques would contribute to elucidate the pathophysiology of BRVO.

## Introduction

Branch retinal vein occlusion (BRVO) is the second most common retinal vascular disorder after diabetic retinopathy^1,2^. BRVO can cause visual impairment due to secondary macular edema, macular ischemia, and vitreous hemorrhage. The clinical course of patients with BRVO varies and there are cases in which macular edema relapses even after various treatments^3^. In addition, some patients may develop neovascularization of the retina, resulting in vitreous hemorrhage^4^. The reasons for the varying course of BRVO eyes have not yet been fully understood. Accordingly, new approaches to elucidate the pathogenesis of BRVO, which have not been clarified by existing methods, may be useful in developing new methods of treatment or assessment of the BRVO.

Blood flow changes in not only the retinal veins but also the retinal arteries occur in eyes with retinal vein occlusion (RVO)^5,6^. Pulsatility index (PI) obtained by Doppler flowmetry is used as an indicator of retinal vascular resistance in evaluating retinal blood flow^7–9^. There are several reports showing increased resistance in the central retinal artery in eyes with central retinal vein occlusion (CRVO) by using PI^5,10,11^. However, there are few studies using PI in eyes with BRVO. Moreover, there has been no visual representation of the index of the resistivity in the retinal vessels. Therefore, it is unclear whether the increased resistance of the retinal arteries is in the localized retinal area where RVO develops or in whole retina caused by inflammatory cytokines or other factors. To elucidate the localization of resistance of the retinal arteries would enable a new therapeutic target or evaluation index for RVO. Therefore, it should be important to examine the resistance of retinal arteries in detail in BRVO eyes.

The laser speckle flowgraphy (LSFG; Softcare Co., Ltd., Fukutsu, Japan) can simultaneously measure and visualize many blood vessels in a noninvasive manner compared to conventional devices, and there are many studies regarding new insights on retinal vein occlusion using LSFG^6,12^. Mean blur rate (MBR) is a value known to represent blood flow velocity in LSFG. In addition, various analyses have been performed on the waveform parameters of MBR^13^. Matsumoto et al. reported that total capillary resistance (TCR), a parameter of LSFG, which is the index of resistivity of the retinal vessels calculated in the same manner as PI, was higher in eyes with CRVO^14^. They suggested that blockage of retinal veins may cause high resistivity in the retinal vessels. However, there have been no studies comparing the resistivity of retinal arteries in the area affected by BRVO and that in the unaffected areas. Beat strength over MBR (BOM) is a new parameter of LSFG that is calculated by the same way as TCR. TCR is measured in the vascular area of the optic nerve head, whereas BOM is measured in retinal vessels. The names of the parameters are different, but both parameters represent resistivity of the retinal vessels.

In this study, we examined vascular resistance in the affected and unaffected areas in eyes of patients with BRVO using BOM. This study aimed to evaluate the resistivity of retinal arteries using BOM and visually represent it as a two-dimensional map, like fundus photographs, using a new technique of LSFG.

## Methods

### Ethics statement

This was a retrospective cross-sectional, single-center study. The Ethics Committee of Nagoya University Hospital (Nagoya, Japan) approved the procedures, and the procedures conformed to the tenets of the Declaration of Helsinki. Informed consent was obtained from all participants after explaining the nature and possible complications of the study.

### Subjects and testing protocol

We reviewed the medical records of the patients with BRVO who received Nagoya University Hospital from December 2013 to May 2020. The subjects with treatment-naive BRVO recorded LSFG of both the BRVO affected eye and the fellow eye were included. Also, healthy patients without systemic diseases such as hypertension and diabetes who were age-matched with BRVO patients and who have cataracts, anterior retinal membranes, macular holes, and retinal detachment in one eye were reviewed, and the fellow eyes of them were studied as control eye. All the patients had comprehensive ophthalmologic examinations, including measurements of the blood pressure (BP) with an automatic sphygmomanometer (CH-483C; Citizen, Tokyo, Japan), best-corrected visual acuity and intraocular pressure (IOP), slit-lamp biomicroscopy, indirect ophthalmoscopy, wide-field fundus photography (Optos 200Tx, Optos plc, Dunfermline, Scotland, UK), and LSFG. The Spectralis^®^ SD-OCT instrument (Heidelberg Engineering, Heidelberg, Germany) was used to measure central foveal thickness (CFT) defined as the thickness between the surface of the inner limiting membrane and the outer border of the retinal pigment epithelium centered on the fovea in horizontal cross-sectional image of BRVO eyes. The extent of retinal hemorrhage in the fundus photograph was defined as the ratio of the range of retinal hemorrhage to the range of optic disc measured by Image J, and it was expressed the unit of disc diameter (DD) in this study. Major BRVO was defined as occlusion of vein extending beyond the arcade vessels to the retinal periphery, while macular BRVO was defined as occlusion limited to a smaller venous tributary located within the arcade^15^.

### Exclusion criteria

Eyes were excluded if they had other retinal diseases, such as macular degeneration, macular hole, vascular occlusive disease, or diabetic retinopathy. Furthermore, we excluded eyes with poor images of retinal vessels on fundus photography and LSFG due to opacity of the optic media and massive hemorrhage around the vessels and eyes in which the vessels to be measured by LSFG could not be recognized because blood vessels run adjacent to each other without gaps. We also excluded eyes that underwent treatment before recording LSFG and eyes with CRVO, hemi-CRVO, or bilateral BRVO.

### Examinations of the retinal artery and vein on LSFG

**Figure 1:**
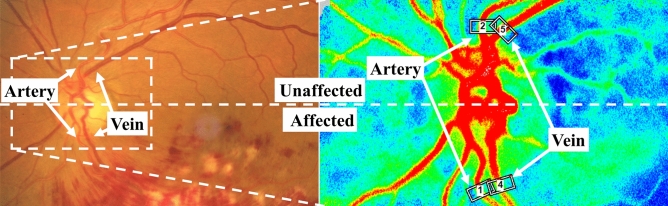
Selection of the arteries and veins of the affected retinal area of BRVO and unaffected area. The rubber bands were placed on the peripapillary retinal arteries and veins running to the affected area of BRVO and also on retinal vessels of the unaffected area, which is vertically symmetrical with them.

Based on the fundus photograph of eyes with BRVO, we divided the retina into two hemispheres, the affected and unaffected hemisphere of the eyes with BRVO. Then, the fellow eyes were also divided into the affected and unaffected hemispheres to be same side as the BRVO eyes. In addition, we made a pair with a BRVO case of similar age of the age-matched control eye and divided the control eye into the affected and unaffected hemispheres to be the same side as the BRVO eyes. Then, we placed the rubber band of LSFG on one retinal artery and one retinal vein running toward the area where BRVO developed. Rubber bands were also placed on the retinal artery and retinal vein in the unaffected retina symmetrical to the locations of the rubber band of the affected area (Fig. 1). Even in the fellow eye and the control eye, the four rubber bands were set in the similar position as the eyes with BRVO.

### LSFG parameters

Detailed reports on the principle of LSFG have already been done^16–18^. In the measurements of retinal blood flow using the LSFG-NAVI system, the MBR, which represents the blood flow velocity, is obtained over several cardiac cycles for 118 frames (4-s period). To evaluate the retinal blood flow of the specified vessels, a rubber band was set on the peripapillary vessels. The system can distinguish between the retinal artery and vein. The MBR in the retinal vessels is calculated by automatically subtracting background MBR from the underlying choroid. The calculation of the relative flow volume (RFV) has been described in detail^19^. The RFV represents the blood flow volume of the retinal vessels. The vessel diameter was determined and used for RFV calculation.

Beat strength (BS) is a parameter provided by the device manufacturer that represents the range of variability of dynamic blood flow changes synchronized with the heartbeat obtained from the region of interest. The blood flow waveform of the time series has variations in each cardiac cycle. It is calculated as a value proportional to the difference between the maximum and minimum values of the individual blood flow waves (i.e., the amplitude of the MBR) by extracting the frequency components that match the cardiac cycle. The detailed calculation method has been published as a patent (https://patentscope2.wipo.int/search/en/detail.jsf?docId=WO2018003139)^20^.

Using this method, the BS can be calculated by analyzing the power spectrum predicted from the short blood flow measurement time of about 4 s in LSFG.

**Figure 2:**
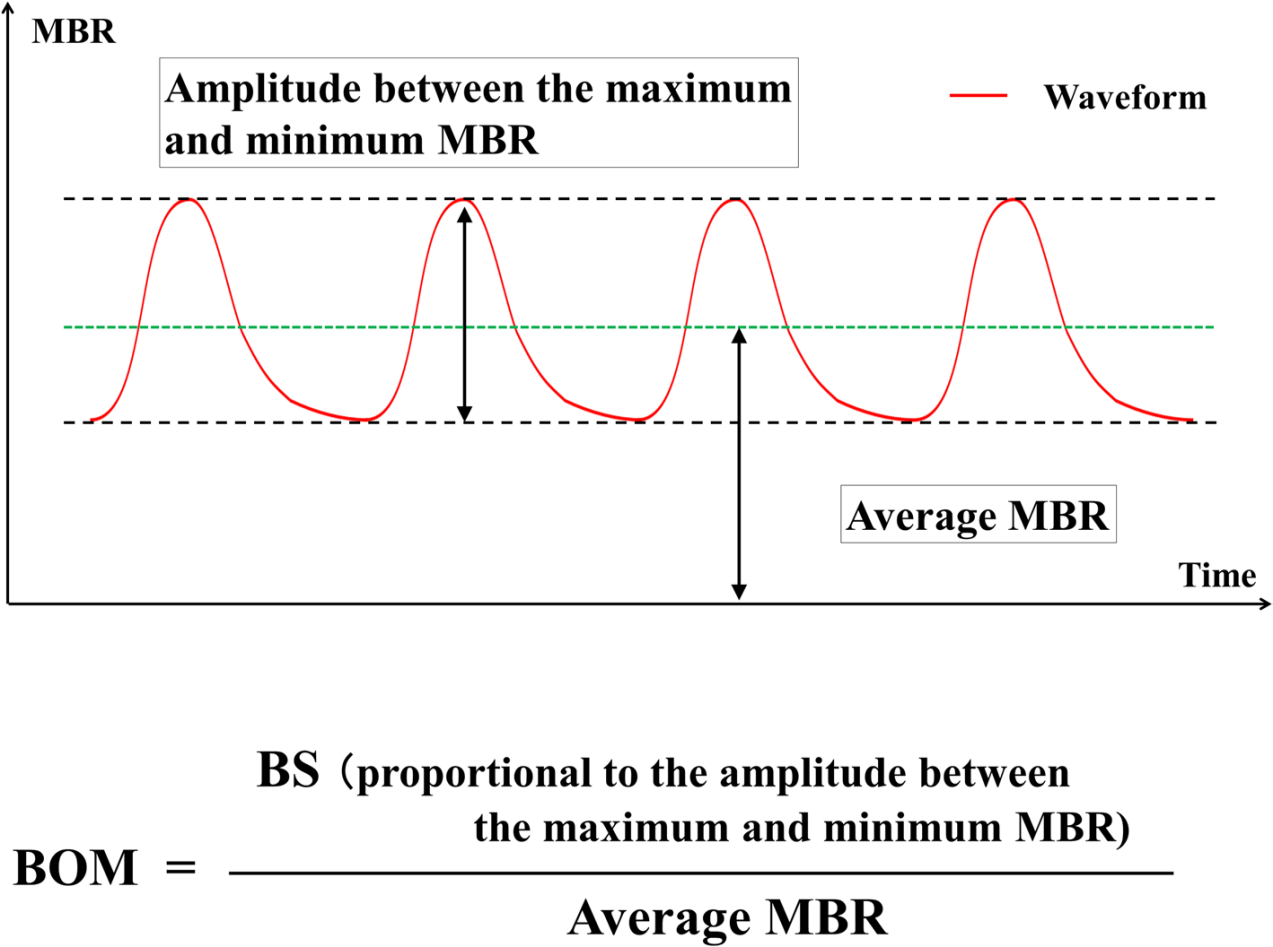
Schematic diagram of beat strength over mean blur rate (BOM). Beat strength (BS) is proportional to the amplitude between the maximum and minimum blood flow. BOM is BS divided by average MBR.

The average blood flow velocity was represented as the average MBR in the specified region. The resistivity parameter BOM is defined as the following equation (Fig. 2):

$${\text{BOM}} = {\text{BS/Average}}\;{\text{MBR}},$$

The arteries of BRVO eyes were examined independently by two retina specialists in a masked manner to evaluate inter-observer reproducibility of BOM. Intraclass correlation coefficient (ICC) was used to evaluate measurement reproducibility of BOM.

### Visualization of waveform parameters of LSFG

For representing vascular resistance of the retina, we used a new software embedded in LSFG. Simple linear iterative clustering, an algorithm used in the field of image processing, groups similar pixels into superpixels, which are structures larger than pixels^21^. Then, it converts the waveform values of each superpixel unit into a two-dimensional map, which provides an overview of the blood vessels and tissue mass.

### Statistical analyses

All statistical analyses were performed with the IBM SPSS Statistics for Windows, Version 26.0 (IBM Corp., Armonk, NY, USA). Chi-square test, paired *t*-test or one-way analysis of variance were used to compare the baseline characteristics and the parameters of LSFG between BRVO patients and healthy control patients, or three types of the eyes. Parameters of retinal blood flow between the affected area of the retina and unaffected area were compared with paired *t*-test. Since the parameters of LSFG are not suitable for comparison between individuals, the ratio of the BOM affected area to the unaffected area of the affected eye was used for considering factors related to BOM. Paired *t*-test was used for comparing the ratio of them between eyes with BRVO and the fellow eyes or the control eyes. *T*-test was done to compare major BRVO eyes with major BRVO eyes for comparing the ratio of them. Spearman’s rank test was used to determine the correlation coefficients between the variables. Multiple regression analysis was used to determine independent factors affecting the ratio of the BOM of the retinal artery in the affected retinal area of BRVO to unaffected. The data are presented as the means ± standard deviation of the means. A *P* value < 0.05 was taken to be significant.

## Results

A total of 39 patients with treatment naïve BRVO were examined for LSFG measurements in both eyes. Three patients with the BRVO on both eyes and four patients with a poor image of LSFG on either BRVO eyes or fellow eyes were excluded. Thirty-two BRVO eyes and 32 fellow eyes in patients with BRVO were enrolled in the analysis. Also, 32 fellow eyes of age-matched healthy patients without systemic disease with cataract, epiretinal membrane, macular hole, or retinal detachment were analyzed. The clinical characteristics of the subjects are shown in Table 1.

**Table 1:** Characteristics of subjects.

Characteristic	BRVO eye	Fellow eye	Control eye	*P* value
*n* (eyes)	32	32	32	–
Sex (male/female)	18/14		15/17	0.453
Age (year)	64.8 ± 11.7		64.9 ± 11.6	0.958
Systolic blood pressure (mmHg)	156.3 ± 16.6		126.0 ± 15.4	< 0.001
Diastolic blood pressure (mmHg)	92.8 ± 11.1		73.5 ± 10.2	< 0.001
Mean arterial pressure (mmHg)	114.0 ± 11.3		91.0 ± 11.1	< 0.001
Mean ocular perfusion pressure (mmHg)	61.8 ± 7.0	61.5 ± 7.2	47.7 ± 8.8	< 0.001
Hypertension	14		0	< 0.001
Dyslipidemia	5		0	0.004
Diabetes mellitus	1		0	1.000
History of smoking	17		6	0.053
Axial length (mm)	23.8 ± 1.1	23.8 ± 1.0	24.4 ± 1.8	0.272
IOP (mmHg)	14.2 ± 2.9	14.5 ± 2.9	13.1 ± 3.0	0.092
Visual acuity (LogMAR)	0.56 ± 0.35	0.02 ± 0.07	0.02 ± 0.06	< 0.001
Period from diagnosis (weeks)	2.3 ± 2.3	–	–	–
Central foveal thickness (μm)	593 ± 187	–	–	–
Extent of retinal hemorrhage (disc areas)	58.7 ± 2.9	–	–	–

**Figure 3:**
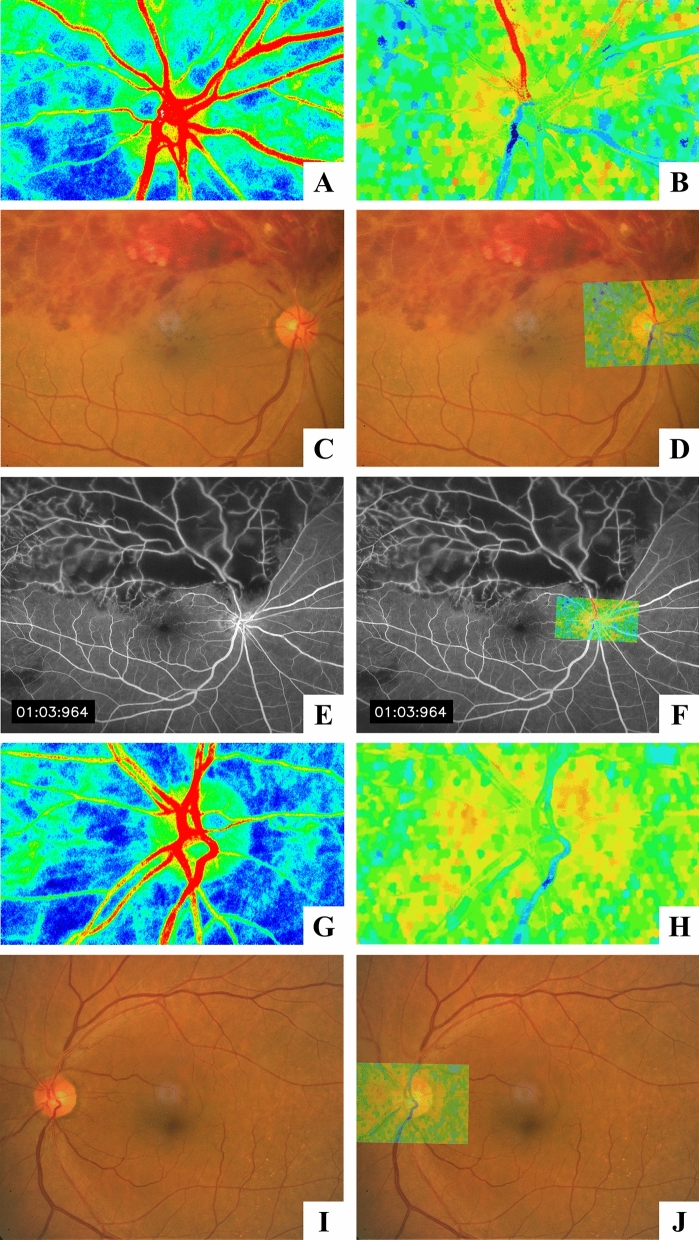
A representative case of major BRVO in the superotemporal retina. (**A**) Composite color maps using the MBR as measured by laser speckle flowgraphy. The red color indicates high MBR, and the blue color indicates low MBR. (**B**) The BOM map visualized by laser speckle flowgraphy shows higher values of BOM in the retinal artery (red color) running to the affected retinal area. (**C**,**D**) Fundus photograph of the BRVO-affected eye and superimposed image with BOM map. (**E**,**F**) Fluorescein angiogram and superimposed image with BOM map showing nonperfusion of the affected area. (**G**,**H**) Composite color maps using MBR and BOM in the fellow eye. There were no high BOM values of the blood vessel in the fellow eye. (**I**,**J**) Fundus photograph of the fellow eye and superimposed image with BOM map.

**Figure 4:**
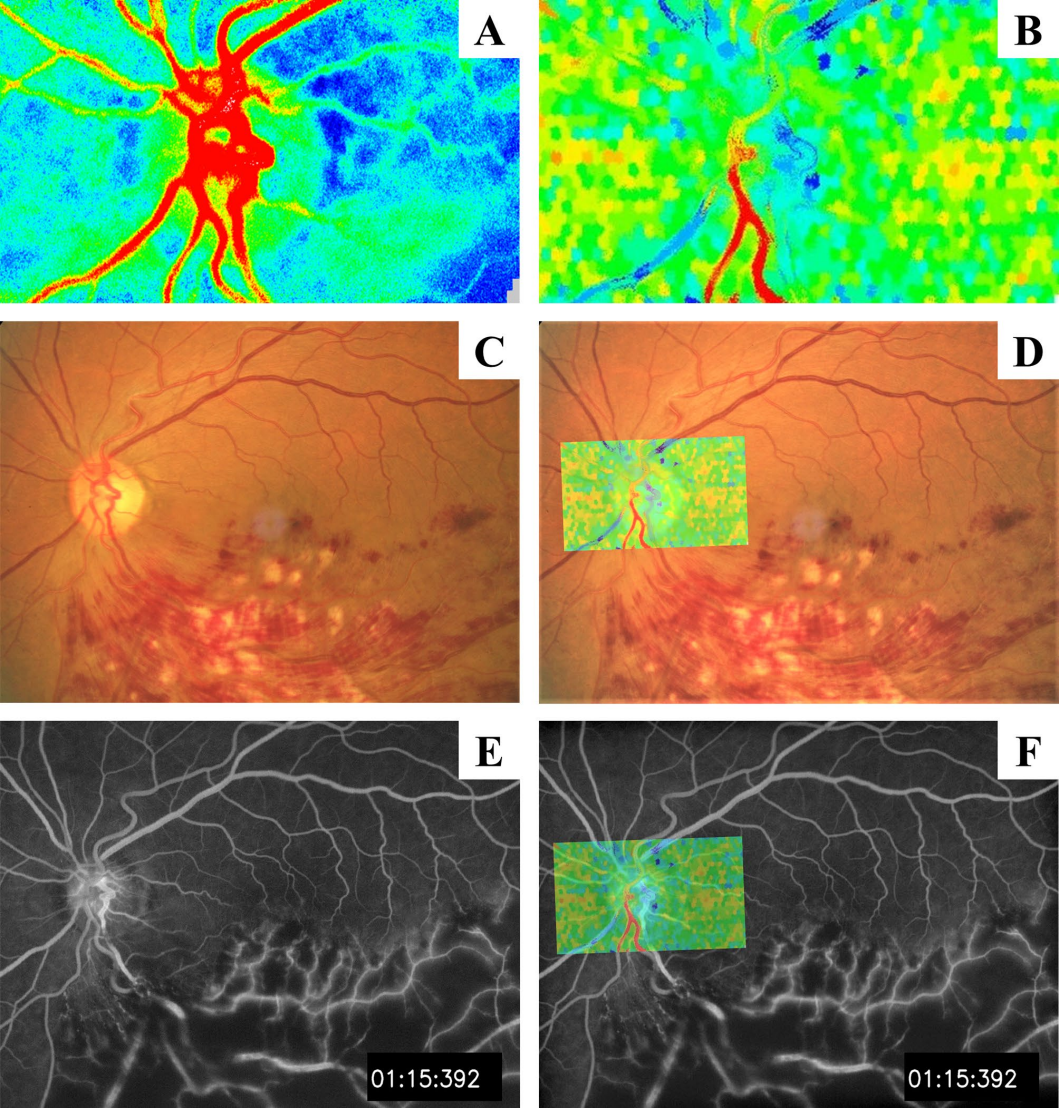
A representative case of major BRVO in the inferotemporal retina. (**A**) Composite color maps using the MBR. (**B**) Composite color maps using BOM shows higher BOM values in the retinal artery running to the affected retinal area. (**C**,**D**) Fundus photograph of the BRVO-affected eye and superimposed image with BOM map. (**E**,**F**) Fluorescein angiogram and superimposed image with BOM map showing nonperfusion of the affected area.

Representative images of LSFG, fundus photography, and fluorescein angiography are shown in Figs. 3 and 4. In the BOM map, it can be visualized that the artery running toward the area of BRVO has a higher value of BOM than the vessels of the other areas. The parameters of LSFG of each area of the retina are shown in Table 2.

**Table 2:** Parameters of the vessels measured by LSFG.

Parameter	BRVO eye			Fellow eye			Control eye		
	Affected side	Unaffected side	*P* value	Affected side	Unaffected side	*P* value	Affected side	Unaffected side	*P* value
**Artery**									
BOM (AU)	1.53 ± 0.63	1.14 ± 0.39	0.001	1.12 ± 0.44	1.11 ± 0.39	0.872	1.27 ± 0.41	1.25 ± 0.39	0.751
MBR (AU)	21.1 ± 7.4	23.8 ± 8.4	0.079	27.2 ± 9.5	25.8 ± 8.4	0.411	20.5 ± 6.7	20.2 ± 5.9	0.833
Vessel diameter (AU)	12.9 ± 2.1	12.9 ± 2.6	0.934	13.2 ± 2.1	13.4 ± 2.7	0.532	12.4 ± 2.1	12.8 ± 2.5	0.508
RFV (AU)	273 ± 114	309 ± 121	0.125	358 ± 135	347 ± 127	0.623	255 ± 95	257 ± 90	0.922
**Vein**									
BOM (AU)	0.79 ± 0.57	0.55 ± 0.22	0.011	0.57 ± 0.28	0.62 ± 0.29	0.145	0.62 ± 0.21	0.66 ± 0.30	0.364
MBR (AU)	22.5 ± 10.2	26.5 ± 7.2	0.026	30.4 ± 8.9	29.0 ± 9.1	0.338	25.8 ± 7.9	23.8 ± 8.0	0.253
Vessel diameter (AU)	14.5 ± 3.2	16.9 ± 3.1	0.002	14.7 ± 3.4	14.0 ± 3.4	0.320	15.2 ± 2.3	16.1 ± 2.7	0.169
RFV (AU)	336 ± 181	452 ± 165	0.002	454 ± 175	422 ± 194	0.288	397 ± 151	383 ± 137	0.694

**Figure 5:**
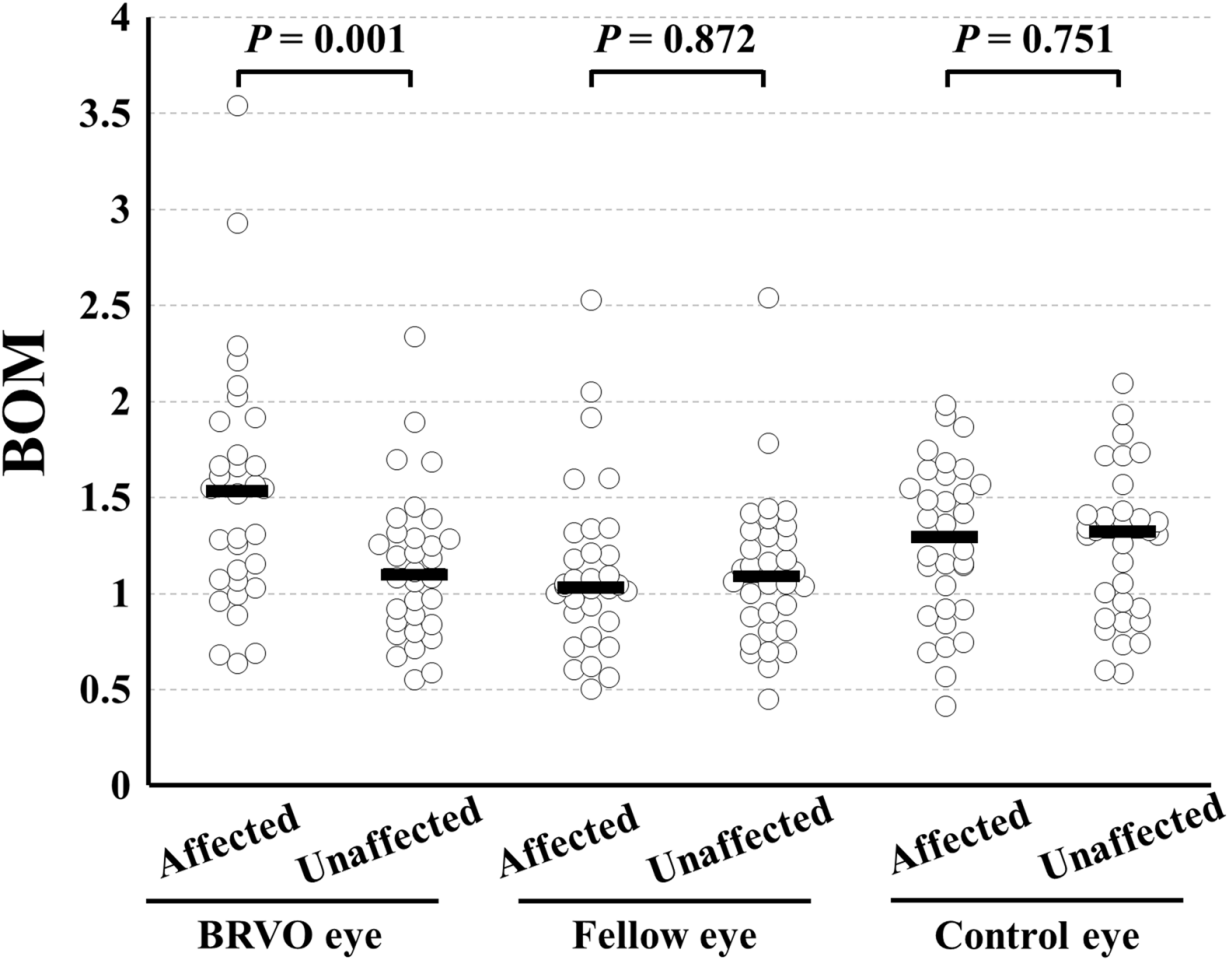
Differences in the BOM of the affected retinal area by BRVO and unaffected area in eyes with BRVO, fellow eyes, and control eyes. In BRVO eyes, the BOM in the arteries in the affected area was significantly higher than that in the unaffected area (*P* = 0.001). In the fellow eyes and control eyes, there was no significant difference between the affected and unaffected areas.

ICC for the BOM of the arteries was 0.959. The BOM of the arteries in the affected area of BRVO eyes was significantly higher than that of arteries in the unaffected area (*P* = 0.001; Fig. 5). In the fellow eyes of patients with BRVO and the control eyes, there was no significant difference in BOM of the arteries between the affected and unaffected areas. Although parameters except for BOM, that is, MBRs, vessel diameter, and RFV, of the arteries in BRVO eyes were not significantly different, BOM, MBR, vessel diameter, and RFV of the veins only in BRVO eyes were significantly different between the affected and unaffected areas (*P* = 0.011, *P* = 0.026, *P* = 0.002, and *P* = 0.002, respectively).

**Figure 6:**
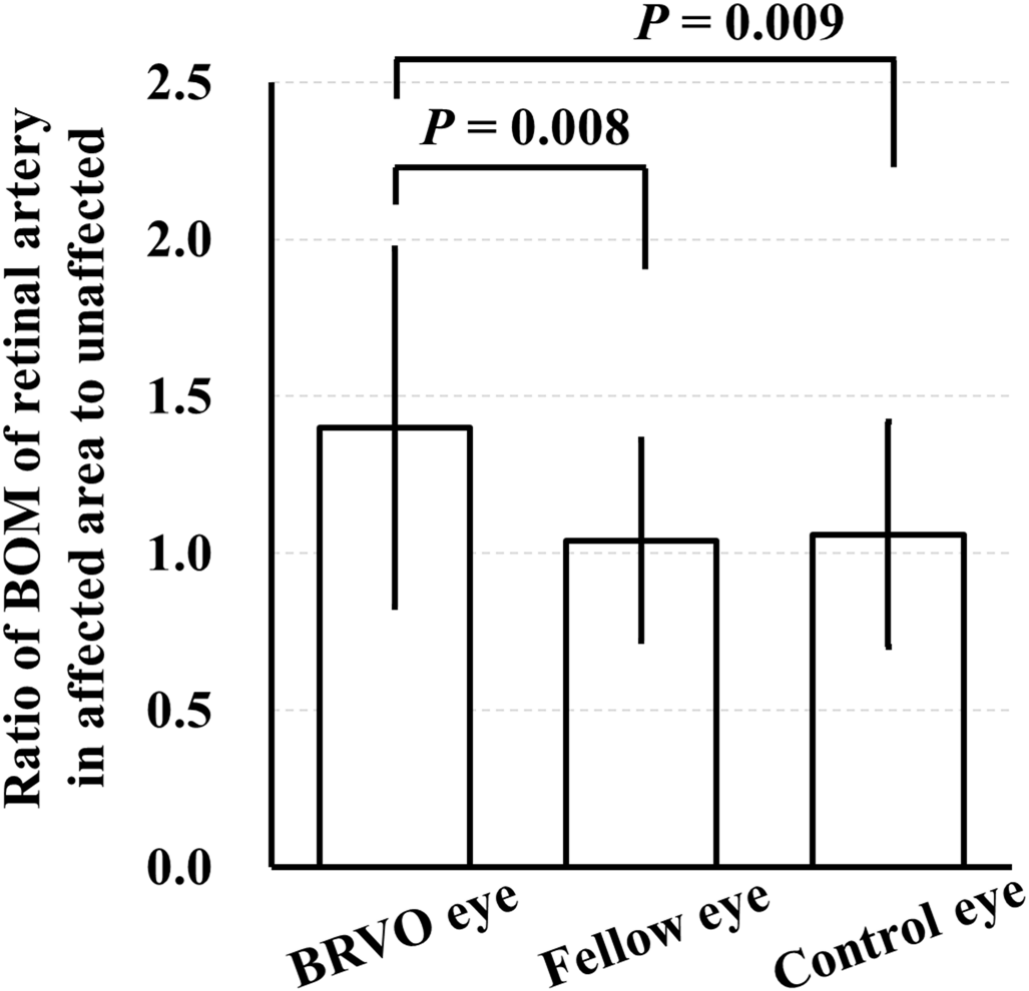
The ratio of BOM of a retinal artery in BRVO-affected retinal area to unaffected area was significantly higher in the BRVO eyes than that in the fellow eyes and control eyes (*P* = 0.008, *P* = 0.009, respectively).

**Figure 7:**
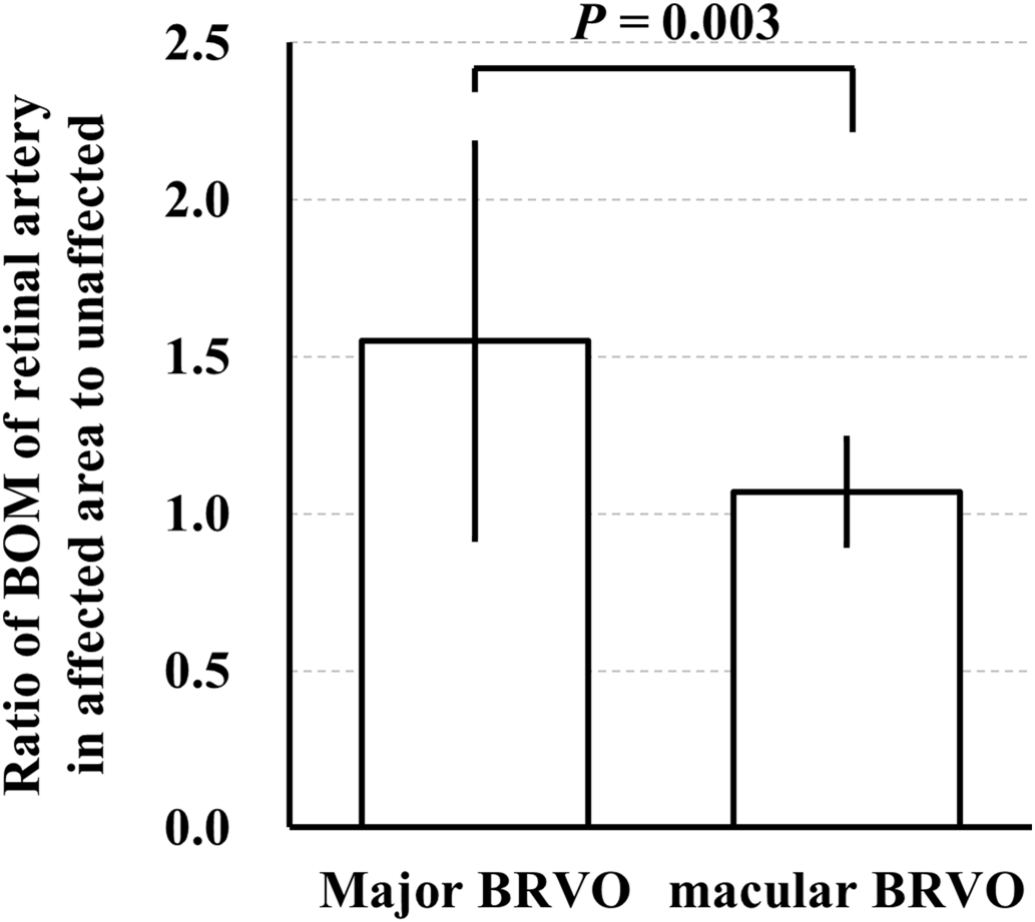
The ratio of BOM of a retinal artery in BRVO-affected retinal area to unaffected area was significantly higher in the major BRVO eyes than that in the macular BRVO eyes (*P* = 0.003).

**Figure 8:**
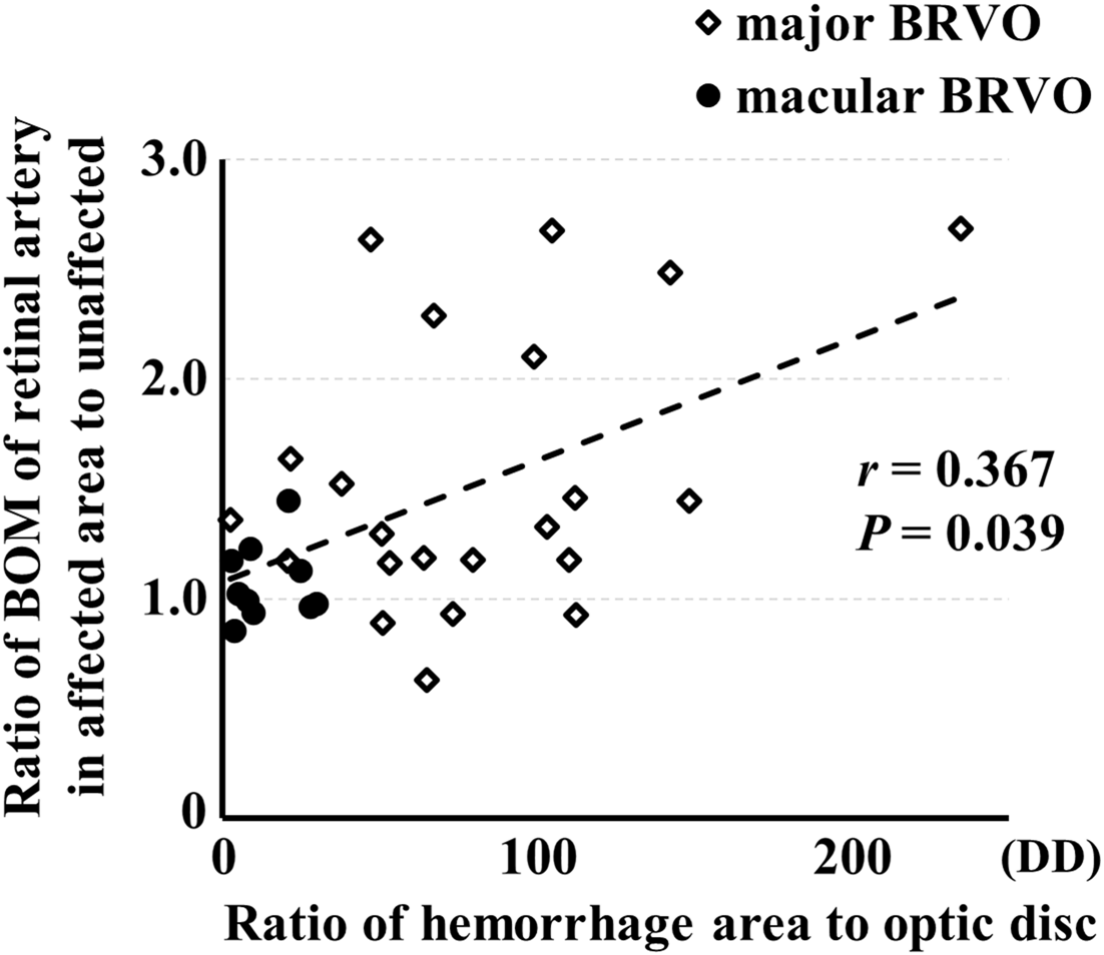
The ratio of BOM of a retinal artery in BRVO-affected retinal area to unaffected area was significantly correlated with the extent of retinal hemorrhage (*r* = 0.367, *P* = 0.039).

The ratio of BOM of the retinal artery in BRVO-affected retinal area to the unaffected area in BRVO eyes was significantly higher than that in fellow eyes and control eyes (*P* = 0.008, *P* = 0.009, respectively; Table 3, Fig. 6). The number of major BRVO was 22 eyes, and the number of macular BRVO was 10 eyes. The ratio of BOM of the retinal artery in BRVO-affected retinal area to the unaffected area was significantly higher in eyes with major BRVO than that in eyes with macular BRVO (*P* = 0.003; Fig. 7). Spearman’s rank test showed that the ratio of BOM of the retinal artery in BRVO-affected retinal area to the unaffected area was significantly correlated with the extent of retinal hemorrhage (*r* = 0.367, *P* = 0.039; Table 4, Fig. 8). Multiple regression analysis showed that the ratio was significantly associated with the extent of retinal hemorrhage (*β* = 0.447, *P* = 0.009; Table 5).

**Table 3:** Ratio of the parameters of arteries in affected and unaffected side.

Parameter	Ratio of the parameters of arteries in affected and unaffected side			
	BRVO eyes	Fellow eyes	Control eyes	*P* value
BOM (AU)	1.40 ± 0.57	1.04 ± 0.32	1.06 ± 0.36	0.010
MBR (AU)	0.94 ± 0.34	1.12 ± 0.40	1.08 ± 0.44	0.164
Vessel diameter (AU)	1.03 ± 0.27	1.01 ± 0.18	1.00 ± 0.27	0.872
Relative flow volume (AU)	0.98 ± 0.56	1.14 ± 0.53	1.09 ± 0.58	0.534

**Table 4:** Results of Spearman's rank test analysis.

Variable		*r*	*P* value
Dependent	Independent		
Ratio of BOM of retinal artery in BRVO affected retinal area to unaffected	Extent of retinal hemorrhage	0.367	0.039
	Central foveal thickness	− 0.340	0.057
	Ratio of BOM of retinal vein in BRVO affected retinal area to unaffected	0.312	0.082
	Visual acuity	− 0.184	0.313
	Period from onset	− 0.178	0.337
	Sex	− 0.177	0.331
	Axial length	− 0.170	0.474
	Vessel diameter	0.087	0.635
	Age	− 0.045	0.805
	Mean arterial pressure	− 0.020	0.914
	Mean ocular perfusion pressure	0.016	0.932

**Table 5:** Results of multiple regression analysis for independence of factors contributing to ratio of BOM of retinal artery in BRVO affected retinal area to unaffected.

Variable		β	*P* value
Dependent	Independent		
Ratio of BOM of retinal artery in BRVO affected retinal area to unaffected	Extent of retinal hemorrhage	0.447	0.009
	Ratio of BOM of retinal vein in BRVO affected retinal area eye to unaffected	0.320	0.053
	Axial length	− 0.125	0.441
	Age	− 0.119	0.465

## Discussion

The BOM map clearly visualized the higher BOM of the retinal arteries in the BRVO-affected area than that in the unaffected area. BOM of the retinal artery in the BRVO-affected area was significantly higher than in the unaffected area in BRVO eyes. Furthermore, the ratio of BOM of the retinal artery in the BRVO-affected retinal area to the unaffected area was significantly associated with the extent of retinal hemorrhage.

Several studies using color Doppler imaging showed an increase in the resistance index(RI) and PI of the central retinal artery and ophthalmic artery in eyes with CRVO compared to control eyes^5,10,11^. However, there are few studies on arterial vascular resistance in BRVO. Yoshida et al. reported that the eye of a patient with single BRVO had high arterial pulsatility, which indicated elevated distal resistance in the affected area^22^. Some studies reported that there was no difference in the resistance index of the central retinal artery measured by Doppler imaging devices between the BRVO eyes and fellow eyes^5,23^. However, these studies compared the central retinal arteries of BRVO and fellow eyes and did not compare the affected and unaffected areas in the BRVO eye; thus, the detail of blood flow situation in the eye with BRVO is still unknown. One of the reasons is the difficulty in simultaneous measurement of retinal blood flow in a short time in previous studies of retinal blood flow using devices, such as Doppler flowmetry. Conversely, LSFG can simultaneously evaluate the blood flow parameters, including BOM, on multiple retinal vessels in a short time.

In this study, we compared the BOM, which shows resistance of the retinal vessel, in retinal arteries between affected and unaffected sites in BRVO eyes. Moreover, we also compared the BOM of retinal arteries of fellow eyes and control eyes in the same area as the BRVO eyes. As a result, the BOM of the retinal arteries running in the affected area of BRVO eyes was significantly increased compared to the unaffected area, and there was no difference between them in the fellow eyes and control eyes. It suggested that the resistance of the retinal artery of BRVO eyes was increased due to BRVO. These results are consistent with previous studies on increased retinal artery resistance in eyes with CRVO^5,10,11,14^.

The possible cause of increased vascular resistance in the arteries of the BRVO-affected area is inadequate circulation in the peripheral retinal vessels. In a report that measured capillary blood flow using scanning laser Doppler flowmetry, the blood volume, flow, and velocity were significantly decreased in the area in BRVO eye compared to the same area in the fellow eye^24^. Moreover, it may be related to changes in the oxygen saturation in the arteries of the BRVO-affected area. Using retinal oximetry, Hardarson and Stefansson reported that the oxygen saturation of arteries in areas affected by BRVO was higher than in unaffected areas^25^. In other studies, the oxygen saturation in areas affected by ischemic BRVO was higher than in unaffected eyes, and the central SaO_2_-A in non-ischemic BRVO was also significantly increased compared to healthy eyes^26,27^. Congestion of venous blood flow may affect the capillary circulation and dynamics of the arteries. If there is ischemia in the affected area, it may have some effect on arterial blood flow due to reduced oxygen demand in that area. However, this study did not assess retinal ischemia, so further research is needed. Thus, the increase in the vascular resistance of the retinal artery was thought to be secondary to the blood flow disturbance caused by BRVO. Furthermore, it is most likely that the ratio of BOM of the affected area to the unaffected area was significantly higher in major BRVO than that in macular BRVO and correlated with the extent of hemorrhage in the present study, indicating that the greater the extent of such retinal blood flow disturbance, the greater the vascular resistance.

In this study, there was a decrease in blood flow and blood velocity in the retinal veins, which is in accordance with the results previously reported^6,22^. Moreover, it has been reported that retinal venous pressure is increased in BRVO, which is consistent with the present results in parameters of veins^28,29^.

The strength of our study is that the BOM in a two-dimensional map, like a fundus photograph, enables clearly visualization of the increase in resistance. This is the first study to visualize the increased resistance of vessels of retinal disorders. This technique may also be helpful in elucidating the pathophysiology, determining the therapeutic effect, or predicting the prognosis of other diseases that cause changes in ocular blood flow, such as glaucoma, diabetic retinopathy, and uveitis.

The present study has some limitations. First, there have been no reports comparing the RI or PI determined by Doppler flowmetry, which is widely recognized as an index of vascular resistance, with the BOM or TCR determined by LSFG. It is necessary to compare those parameters using Doppler flowmetry and LSFG in future studies to evaluate the relevance. Second, the sample size was small (32 eyes of 32 patients). Third, the analysis was conducted only at the time of the initial diagnosis and did not analyze the subsequent time course of the disease. Fourth, we did not measure substances, such as VEGF, in the eyes, which may have affected the results. Fifth, we did not evaluate ischemia area using fluorescence fundus angiography. Eyes with ischemic areas may show different hemodynamics from eyes without ischemic areas. Sixth, there were differences between the values of the LSFG parameters of the control eyes and fellow eyes of BRVO. However, we did not compare the values determined by LSFG among the groups in this study because it is still controversial to compare the values among individuals. Therefore, we used the ratio of LSFG parameters in the affected area to the unaffected area to compare among groups, which is described in Table 3. Lastly, the courses of patients with BRVO are different, but it is still unclear how our results are related to visual acuity prognosis or treatment responsiveness. However, our findings have possibilities of a novel factor in predicting the prognosis of patients with retinal vein occlusion or indicator of treatments. Further prospective studies on a larger number of eyes with more detailed information of change of function and morphology will be necessary to evaluate the possibility of our findings determined by the new parameter BOM.

In conclusion, a new parameter BOM clearly visualized that retinal arteries in the affected area by BRVO had higher resistance and showed that the degree of the resistance was significantly associated with the extent of retinal hemorrhage. The finding would contribute to the elucidation of pathology in retinal disease, including BRVO, in the future.
